# An Anatomical Description of the Diseases of the Organs of Circulation and Respiration

**Published:** 1847-02

**Authors:** 


ARTICLE IX.

*An Anatomical description of the Diseases of the Organs of Circulation and Respiration.* By Charles Edward Hasse, Professor of Pathology and Clinical Medicine in the University of Zurich, etc. etc. Translated and edited by E. W. Swayne, M.D., Physician Extraordinary to H. R. H. the Dutchess of Kent. Philadelphia: Lee & Blanchard. 1846. pp. 277. 8vo. (From the Publishers.)

Pathological Anatomy is the only sure foundation upon which those important parts of the fabric of medical science, diagnosis and pathology can rest. If there are any who doubt its value, it must be from not having sufficiently reflected upon the history of medicine, or noticed with attention the obstacles that have most opposed its progress. The earlier medical writers excelled in minute observation and description of symptoms. They sketched the portrait of disease with a master's hand, but for want of a knowledge of the changes which the organs undergo, their descriptions are comparatively useless at the present day. How are we to know whether the cases they describe as consumption were not bronchitis? What assurance have we that their typhus fevers of old persons, and occurring in winter, were not pneumonias? Certainly none whatever; and this fact is so well known that recent authors have found former observations so little to be depended upon, that they have determined to reject them altogether, and commence anew the acquisition of facts which may be relied upon as authentic. We have but to look at those departments of medical science in which much progress has been made, and the services rendered by pathological anatomy will be apparent.

Diseases of the brain, of the heart, inflammation of the veins, and many others of obscure character, have been rendered familiar by post mortem examinations. By the term pathological anatomy we do not mean simply the changes left in an organ by disease which has resulted fatally, but a history of the changes of structure which an organ undergoes during the progress of the malady—in other words, an *anatomical* history of disease.

Such is the view of our author, who possesses ample knowledge and means for doing justice to the subject, having for a length of time pursued his researches at Paris, in a school which numbers among its teachers such names as Andral and Louis. This work, without being a compilation, embraces the most important outlines of the science as developed by others, but in several chapters exhibits originality, and in others a candid and thorough criticism which mark a superior mind. We shall not attempt to follow him through his description of the lesions of the circulating and respiratory organs, but prefer to give, as samples of the work, a few extracts, selecting such as from their originality possess public interest.

Of this kind will be found the following section upon an obscure affection.

"A product of inflammatory action deserving of notice here, are the *fibrinous coagula* occurring within the cavities of the heart. Formerly much importance was attached to these so-termed polypi of the heart, and no small disposition was evinced to regard them, not only as the immediate agent of death, but likewise as the main source of many chronic diseases of the respiratory and circulating organs. Pasta combated this extravagant notion, but went too far in asserting that every polypoid formation within the heart was the result of stagnation of the blood after death, and that even fibrinous depositions within aneurismal sacs were impossible during life. Afterwards, when organic diseases of the heart were more minutely studied, fibrinous coagula became the subject of repeated research, and it is Kreysig's merit to have shown that, although they could not be looked upon as either the cause or the produce of chronic disease, they yet might be the result of an inflammatory process affecting the inner surface of the heart; in short, that a polypous carditis must be recognized. Bouillard arrives at the same conclusions, but, on the other hand, perhaps assigned undue weight to endocarditis in the production of polypi.

"Of the blood coagula, two types are commonly acknowledged, the distinctions between which are essential and obvious; there are, however, so many transition-forms, that it is often extremely difficult to come to a definite conclusion. The first type is characterized by the simple fibrinous clot, so common in robust individuals. It is yellow, pellucid, of the elasticity of jelly, of a smooth shining surface, and fashioned to the shape of the heart's cavities, especially at the origin of the pulmonary artery, where it often appears moulded with a faint impression of the valves, It seldom fills the cavities however, and never adheres to their parietes. This gelatino-fibrinous mass occasionally covers a clot of coagulated blood, in the same proportion as the buffy coat the crassamentum in blood drawn from a vein; it, however, frequently constitutes the bulk of the concrement, and contains only a few portions striated or dotted blood-red. Polypi of this description, often proceeding with flattened ends into the large vessels, and being usually of greater volume in the right half of the heart than in the left, are developed during the agony, or after death; but result, in no instance, from disease of the encardium. The second type of polypoid coagula is characterized by opaque, white or dingy-grey, soft, filamentous and elastic concrements, consisting of several irregularly superimposed layers with an uneven surface more or less firmly adherent, especially in the neighborhood of the valves, to the heart's parietes, and insinuating their fangs among the columnæ carneæ and tendinous cords. They rarely form a single coherent mass like the foregoing species, but rather unequal and distinct accumulations, connected together through the medium of coagulated blood, or else of a brown or grey pultaceous substance. In rare instances, several of the fibrinous layers include in their centre purulent fluid. At their surface, and even between the different layers, dots and little stripes of blood are discernible. The majority of these coagula are developed during life, and are frequently the *immediate cause* of death. Bouillaud regards them as products of an inflammatory process affecting the encardium, and as being partly secreted by this membrane, partly deposited by the blood while passing over the inflamed parts.

"An unbiased survey of the cases in which these polypi occur, will render it apparent that their development does not necessarily depend on inflammation of the encardium, but that the seat of the morbid action is often, nay, in the majority of instances, remote from the heart. In proving this, the fact should be borne in mind, that certain substances when brought in contact with the blood cause its coagulation, and this the more rapidly, the larger the amount of blood experimented upon, and the more briskly they are agitated and mingled with it. According to the experience of most pathologists, such substances are met with in the blood itself,—tubercle, for instance, cancerous matter, and more frequently pus, which last is particularly efficient in promoting coagulation. These entering the circulation by degrees, will not fail, at whatever part of the body engendered, to accumulate eventually within the cavities of the heart. For it is well known that the ventricles do not empty themselves completely during systole, but retain a small portion of blood; this, being by the shock of the heart's contraction impelled into the network of the columnæ carneæ, acquires there, under the circumstances above indicated, the opportunity to coagulate; the same relations subsisting, the coagula, at first small, gradually increases in bulk, so as eventually to impede, and ultimately to obstruct the circulation, and after death to present a fibrinous concrement of the character last described. It follows from the foregoing explanation, that even those polypi which contain fluid pus, are not necessarily the product of endocarditis."

"CYANOSIS. MORBUS CŒRULEUS.

"Of late years it has been very generally admitted that the cyanotic state is not symptomatic of one, but of various diseases, both of the lungs and heart. Nevertheless, for brevity's sake, we shall here use the term *cyanosis* in its older and familiar acceptation, namely, as denoting a preternatural communication between the right and left compartments of the heart.

"This morbid condition is for the most part dependent upon congenital malformation: as such, however, it is (as clearly demonstrated by J. F. Meckel) not to be regarded as merely accidental, but as subject to the same laws which govern the normal development of the organism.

"A preternatural communication between the two chambers of the heart, and the commingling of arterial and venous blood sometimes consequent thereupon, occurs under a great variety of circumstances. In the following pages, however, our attention will be devoted almost exclusively to those forms in which life is capable of considerable prolongation subsequently to birth.

"The first in importance is an unclosed state of the foramen ovale,—a malformation not only the most frequent of those we have to consider, but at the same time almost invariably a complication of all the rest. Incomplete closure of this aperture, admitting, through an orifice at its margin, the passage of a probe or of the handle of a scalpel, in short, imperfect *valvula foraminis ovalis*, is very often unattended by cyanotic symptoms. Out of 155 individuals, none of whom had manifested any such symptoms, Bizot found the foramen ovale more or less open in 44 instances.

"Where, however, the aperture remains destitute of either a sufficient muscular plug, or an adequate valve, secondary disease of the heart, eventually productive of permanent, or at least of fleeting symptoms of cyanosis, has been invariably known to follow. The marginal edges of the unclosed *foramen ovale* are partly tendinous, partly muscular, sometimes of almost callous hardness. In certain instances the valve or the fleshy plug is variously perforated, the width of the aperture varying from 1½ to 15 Parisian lines.

"The permanence of *Botalli's duct* appears to be rare, and always coincident with unclosed foramen ovale, and with perforation of the septum of the ventricles.

"*A communication between the ventricles*, through an aperture in their septum, may coexist with closure of the foramen ovale, and is sooner or later productive of a cyanotic state. Louis has collected 5 cases of the kind (one, that by Jackson, of the English General Whiple); in a sixth related by Otto (Neue seltene Beobacht, p. 49) of a girl aged 12½, the right ankle was wanting, and the veins of the trunk opened at once into the right ventricle. Perforation of the ventricular septum is more frequently observed in common with a patulous state of the foramen ovale and of Botalli's duct; for example, in the instance so often cited from Richerand's 'Physiology,' of a man aged 40, or in Nasse's interesting case of a girl aged 19

(Leichenöffnungen, p. 162). The aperture in the ventricular septum is mostly situate near the base of the heart, immediately below the origin of the pulmonary artery and aorta; sometimes, however, nearer the apex of the heart, as in Otto's case. This corroborates the opinion of Albert, that the perforation is nearer to the base when the foramen ovale is open, and nearer to the apex when it is closed. The communication between the two ventricles is sometimes direct, sometimes oblique,—duct-like. In the case of a child 13 years old, I once found it crossed by a tendinous band. Its borders are mostly smooth, consisting of fleshy columns: but occasionally, too, of tendinous or cartilaginous hardness. In the cases recorded, the width varies from 2 to 12 lines; it may, however, amount to partial (Bouillaud,—in a child of 4 years) or almost entire (Burdach,—in a youth of 16) deficiency of the ventricular septum.

"The cardiac origen of the great arteries, is liable in like manner to many anomalies, all attended with malformations of other kinds within the heart, mostly proving fatal at an early period under the symptoms of cyanosis, and seldom compatible with prolonged life. In five cases collected by Albers, the pulmonary artery sprung from the left, the aorta from the right ventricle; in all, death ensued within a few months after birth; the two eldest of the children having respectively attained the age of three (Albers) and five (Farre) months. An interesting case is related by Martin, (Müller's Arch. 1839, fasc. 3, p. 222,) in which death occurred after the tenth week. In this state mixed blood circulates, occasioned by the open condition either of the foramen ovale, or of Botalli's duct, or else by an aperture in the ventricular septum.—In two cases of Farre's, (an infant of three weeks, and a male child of eight months,) the pulmonary artery arose from both ventricles. The proper aorta gave off the arteries of the head and of the upper extremities only, and united by a thin branch with the descending aorta, which was derived from the pulmonary artery, beyond the point where the latter sent off its branches to the lungs. The origin of the aorta from both ventricles has been more frequently observed. In seven instances (Louis, Albers, Bouillaud, and Meyer) life was long protracted. The most remarkable examples are, of a girl of 9 years (Cerutti), a boy of 13 (Sandifort), a girl of 17 (Albers), and a woman of 25 (Tommasini); a girl of 17, admirably described by Meyer (Rust's Magazine, vol. lv. fasc. 1, p. 158). In this malformation the pulmonary artery is mostly very narrow, and its semi-lunar valves, like those of the aorta, are either partial or wholly wanting, being faultily fashioned, or rudimental only.

"Are the preternatural communications between the right and left divisions of the heart, just described as the source of cyanosis, always to be regarded as *congenital* malformations? Louis answers this question in the affirmative, and as far as concerns the anormal origin of the great arteries, the coincidence of perforation of the heart's walls with a pervious state of Botalli's duct, and with other malformations acknowledged to be congenital, the thing is indisputable.

"It is otherwise, however, with respect to simple perforation of the ventricular or auricular septa. Bouillaud holds these to be in part the result of ulcerous destruction of the muscular substance with subsequent cicatrization, and refers in particular to a case of Thibert, in which the edges of a perforation of the ventricular septum, in a man aged 24, were furnished with a yellowish membranous fringe. There is, however, no mention made of softening of the vicinity, or of other changes of the encardium, usual in ulcer of the heart; moreover, the foramen ovale was open, and precisely in the condition it was wont to be found in a malformation obviously congenital. Meckel and Abernethy believe in the possibility of the foramen ovale *reopening* in advanced age,—especially in disease of the lung. Otto, who seems of the same opinion, adduces 13 cases of dropsical accumulation within the pleural sacs, with strong adhesion, induration, inflammation, or suppuration of the lungs, (in one instance with coarctation of the pulmonary artery, in another with ossification of the pulmonary sigmoid valves,) in which the foramen ovale was found open; cyanotic symptoms having for a longer or shorter period preceded death. He particularly refers to the case of a man of sixty who had died of hydrothorax, and in whom the right side of the heart and the pulmonary artery were found dilated; the foramen ovale being indeed closed, but its fossa of such depth as to project like a thin pouch into the left auricle. He looks upon this example as demonstrating the way in which gradual reopening of the foramen ovale may take place. It must, however, be borne in mind that the unclosed state of the foramen ovale may, in several of the 13 cases, and especially in those in which the pulmonary artery deviated from natural, have existed from birth, without betraying itself by any decided symptoms, until some disease in the respiratory and circulating organs supervened; and, again, that the cyanotic symptoms may have sprung from the pulmonary affection alone, imperfect closure of the valve of the foramen ovale (which condition Bizot has shown to be no less frequent than insignificant) being merely a coincidence. In a word, although the possibility of acquired perforation of the heart's septa cannot be denied, no positive example of it has been witnessed.

"The relation of the *Eustachian valve* to the unclosed foramen ovale, first hinted at by Wolf, and more closely investigated by Meckel, is not unimportant. Among 80 hearts examined with reference to this point, he found 43 instances of open foramen ovale, with the Eustachian valve large, often muscular, and entire, or but slightly reticular; in a woman of sixty in particular, the foramen ovale was an inch wide, and the Eustachian valve two inches long, almost half an inch high, and very strong. In 16, the foramen ovale was closed, and the Eustachian valve absent altogether, or merely rudimental, or else torn to pieces. In 13 the Eustachian valve was very strong, often muscular,—and the foramen ovale closed. In 8 there was hardly any Eustachian valve, and the foramen ovale was more or less gaping. The frequency of this favorable condition for the passage of venous blood to the foramen ovale and the left auricle, cannot therefore be doubted; and the presence of a strong Eustachian valve may be regarded as conducive to a mingling of venous and arterial blood.

"But what are the necessary conditions to this morbid commixture of the blood? This question is forced upon us by a consideration of the fact, that the influence of the mixed blood upon nutrition, shall be very palpable in some patients, and null in others. Indeed, in many cyanotic subjects the energy of the vital functions is unimpaired, (females menstruate regularly, and procreate healthy children, &c.) while others are constantly ailing, highly sensitive with respect to cold and other external influences, stunted in growth, (many emaciate, others again prone to obesity,) and perish, either early in life, or at one of the periods of evolution. In the former instances, therefore, the passage of venous blood into the arterial, must either not occur at all, or but to a slight extent, or only at certain periods. Jules Cloquet and Louis have shown that, apart from the cases in which a preternatural origin of the blood vessels necessarily keeps up an incessant mingling of the two kinds of blood, it does not take place through an orifice in the septa of the heart, when the walls of the two cavities which communicate with each other, are of equal thickness and strength, and scarcely, even where their strength is unequal, provided the heart's orifices are sufficiently ample. And in reality, wherever there is impaired nutrition, those relations are found wanting in various ways. In most cases there is present hypertrophy of the right auricle and ventricle, mostly eccentrical, but sometimes also concentrical. The walls of the right ventricle are frequently much thicker than those of the left, whence the heart assumes an almost globular shape, and quite a transverse position. Sometimes the left auricle and ventricle are simultaneously hypertrophied, though in not more than a fifth of the cases. The heart is then enormously enlarged, so as to embarrass the neighboring organs more or less, and to deviate from its natural position both behind and below. Under these circumstances, inflammatory phenomena about the heart and pericardium are not unfrequent precursors of death. In six cases described by various authors, the signs of pericarditis, though for the most part of a modified character, were cognizable.

"*Coarctation* of the *right arterial orifice*, which is observed, in a smaller or greater degree, in about one half of the cases, must here exert a most material influence. On some occasions the pulmonary artery, at its commencement, is considerably narrower than usual, and does not dilate, until after the reception of Botalli's duct, in its pervious state; or there are ossifications at its valves, two of which perhaps are alone present; or in place of the valves there is a transverse membranous expansion, with a minus aperture; or the approach to the pulmonary artery within the ventricle, is contracted, forming a small channel, or, as in Nasse's case, blocked up by fleshy columns, hardly separate from each other. In all these instances, it is obvious, that the venous blood is more or less readily impelled directly into the left division of the heart. Nevertheless, there being not rarely a concomitant coarctation of the right auricular orifice, whether simple or arising from preternatural formation, or from ossification of the tricuspid valve, the possibility of arterial blood passing into the right side of the heart, especially during diastole, must be admitted,—more particularly where the right chambers of the heart are at the same time dilated. Upon the whole, this last mode of preternatural admixture of the blood must be acknowledged to be highly influential in determining the hypertrophy and ossifications of the right side of the heart, so common in cyanosis; inasmuch as these morbid conditions are otherwise almost entirely restricted to the left side, which *in cyanosis* is for the most part exempt from them.

"The most remarkable symptom of the organic conditions described, namely, the *blueness of the skin*, was formerly thought to be amply accounted for by the mingling of arterial with venous blood: the simple fact, however, that the decoloration is not permanent through life, deprives the explanation of its weight. The cyanotic phenomena sometimes do not set in until the third, seventh, or fourteenth year, nay, even later,—it may not be until shortly before death; occur periodically only, and at long intervals; are frequently brought on by quick walking, hasty movements, passionate emotions, intercurrent diseases, (hooping-cough, for instance,) external violence, and the like, and thus become permanently established, or else again vanish for a time. If the blueness depended upon the character of the blood, these alternations could not subsist. When, moreover, we reflect that in the fœtus, in which mixed blood always circulates, no such decoloration is perceptible; and that in a case by Breschet, in which the left subclavian arose from the pulmonary artery, no alteration of color was visible in the left arm; we feel it necessary to look for other grounds for an explanation. This was already indicated by Morgagni. Kreysig (Herzkrankheiten, vol. ii.) was, however, the first to form a correct view of the subject, and Louis has subsequently demonstrated that the cyanotic tinge results from impeded flow of the venous blood back to the heart, or from thence to the lungs; any obstacle to the entrance of the blood into the right ventricle, or to its exit from thence into the pulmonary artery, being of course, in a high degree, propitious to such stagnation. It may be inferred from the foregoing, that from the organic relations peculiar to cyanosis, the stagnation in question may increase vastly on the preexisting impediments to the circulation being augmented by accidental causes, whether external or internal.

"Among the morbid phenomena in other parts of the organism, incident to cyanosis, there is not one that is not common to disease of the heart generally, and to hypertrophy in particular. Even the bulbous shape of the distal phalanges of the fingers, and the incurvation of the nails, are no more peculiar to cyanosis than to sundry pulmonary disease. A curious circumstance is the alleged tendency to whitlow among cyanotic individuals. Diminutiveness of the spleen, thyroid gland, and renal capsules, as urged by Nasse, is by no means constant, the reverse having been found in several instances."

### "FŒTAL CONDITION OF THE LUNG AFTER BIRTH.—ATELECTASIS.

"This disease consists in the imperfect expansion of the lungs by the first inspirations after birth; that is,—in a permanence of the fœtal state, of the new-born infant. It is to be regarded as a disease dependent upon restricted functional development at the time of birth, and not upon any original defect of formation in the respiratory organs. When at all extensive, atelectasis terminates sooner or later in death; under more favorable circumstances, however, in early and complete, or in tardy and imperfect recovery. For, during the first days after birth, it is still possible that the evil may be surmounted by the vigorous penetration of air; whereas, at a later period, organic changes supervene, which forever incapacitate the undeveloped portion of lung from performing its proper office. There is, however, still wanting a complete account of atelectasis, as regards both materials and pathological deductions. I shall now endeavor to furnish as faithful an anatomical description as the case will admit of, the result partly E. Jörg's and partly my own research.

"An entire lung, or even an entire lobe, is seldom found in a state of atelectasis,—but for the most part only single and scattered lobules. Experience shows that certain portions of the lungs are especially prone to retain the fœtal condition, namely, the inferior lobes of both lungs, and the posterior half of the remaining lobes generally. Still examples occur of several lobules near the anterior surface being found in like manner undeveloped.

"The diseased patches display a brown-red, or rather blueish-red color, which is more intense if the whole lobule is uniformly unexpanded,—in which case, it is marked off by a sharp contour from the surrounding pale-red healthy substance. Where, on the other hand, scattered cells within such a lobule have become inflated, the violet colour is interrupted here and there, and passes by a gradual transition, and without any distinct boundary, into the natural shade. A distinguishing feature of atelectasis is however this, that the above patches upon the surface of the lungs always exhibit a depression, the superincumbent pleura remaining perfectly smooth and polished. A lobe either entirely, or for the most part, in this condition, is never found enlarged, but on the contrary, of much smaller dimensions than the others, and almost as much collapsed as in the fœtus; in general deeply imbedded within the thorax, and drawn towards the entrance of the bronchi and blood-vessels. Hence single diseased lobules do not attain the same elevation of surface as the healthy ones with which they are surrounded, but, as already stated, form depressions more or less considerable, so that the general aspect may be likened to the dimples created by emphysema, in adult lungs. Neither by incision or pressure is any crepitation produced, unless where a few air-cells happen here and there to have become expanded. The same delicate reddish froth is never found here as in the healthy parts of the lung, but merely a small quantity of serous, slightly sanguineous fluid. The cut surface appears smooth—uniform,—without a vestige of granular elevations. The whole of the diseased structure is not softened, but rather of a hard character, still without the tenacity of the healthy parts. When a patch so situate is cut off and placed in water, it sinks to the bottom.

"When atelectatic infants die a day or two after birth, it is generally possible to dilate, artificially, the undeveloped parts. The depressed lobule is then seen to rise by degrees to the level of the rest, and to assume the color, permeability, and other characters of sound lungs. Up to this point, had other circumstances been favorable, perfect recovery might have taken place. Where, however the little patients have survived for weeks or months, this inflation seldom succeeds, or only imperfectly. At this junction the unexpanded pulmonary cells are for the most part coherent: a remarkable fact, seeing how long the lungs continue unexpanded in the fœtus, without adhesion ever taking place. What ulterior transformations go on in the diseased parts, it is not yet satisfactorily determined; it is, however, more than probable that not a few endurations and depressions, especially the small calcareous concretions, sometimes occuring without any obvious cause, at particular spots within the lungs, (generally at the back of the inferior lobes,) are referrible in some measure to the above source. At all events, it may be observed generally that, in atelectasis, the boundary line between the diseased and the healthy substance becomes less and less distinct, in proportion as life is prolonged. At the earliest period of infantile existence the contrast is very decided, the diseased parts being immediately surrounded by pulmonary texture in all respects natural and healthy.

"In infants who had died of atelectasis, E. Jörg invariably found the foramen ovale of the heart unclosed; a fact confirmed by myself, but which, at that age, is not unusual. The brain was in a congested state. When death followed shortly after birth, the body had the apppearance of being generally well developed, but was extensively ecchymosed; the hands and toes were clenched; and there was foam in front of the nostrils and of the closed mouth. Where, however, the disease had been of some standing, the body was wasted and the skin loose and wrinkled. Jörg remarks, that under these circumstances, both the affected and the adjacent parts are found inflamed, if not in a state of suppuration. The details of Jörg's cases, however, clearly show them to have been complicated. Inflammation is neither necessarily nor even frequently the sequel of atelectasis, for often as I have witnessed this disease, I have never met with a case of inflammation which could be directly traced to the diseased lobule. I have even seen a case of genuine pneumonia with hepatization of the inferior lobe, one portion of which being *atelectatic*, had not participated in the inflammation, but presented hard, knotty, depressed patches in the midst of expanded, softened, hepatized substance. Lobules retaining the fœtal condition, are quite passive in relation to other morbid processes, and especially to inflammation; the examples given by Jörg appear to have been cases of real pneumonia.

"It is here requisite to state that *atelectasis*, with the characteristic features above described, is not always distinguished from other diseases; that condition having, in some instances, not been duly recognized, and, in many more, been confounded with quite dissimilar pulmonary affections. Indeed, the greatest discrepancy of opinion, or rather the greatest confusion prevails, so that I am here under the necessity, however hazardous it may appear, of submitting those discordant views to the ordeal of a critical examination. The French writers on children's diseases, who first turned their attention to the changes in the pulmonary texture of new-born infants, refer *atelectasis* chiefly, if not exclusively, to the head of *pneumonie*

*lobulaire.* Several German authors have, without further inquiry, adopted this view, (Vernon, Der Arat am Krankenbette der Kinder, Wien, 1838, vol. ii. p. 54, &c.;) while others, admitting the peculiarities of atelectasis when associated with ordinary pneumonia, continue nevertheless to connect it, either directly or indirectly, with the same inflammatory process. It will not be difficult to show wherein lies the essential difference between the two. In atelectasis, the coloring of the diseased portions of lung always approaches more to a violet, their exterior appearing smooth and glistening, so as to contrast with the dull, brown-red surface of inflammation. In inflammation, again, the diseased portions are preternaturally distended, while in atelectasis they are collapsed, and inferior even to the healthy texture in volume,—but susceptible, provided the disease has not lasted too long, of artificial inflation, and capable, through its means, of acquiring a perfectly natural appearance. In inflammation, the pulmonary texture is softened, in atelectasis it is hard, and the cut surface is not granular, but smooth. Where no complication exists, the anatomical characters of a first or third stage of pneumonia are not discoverable either in or near the diseased patch; in short, we have nothing like pneumonia except the solid, noncrepitant mass, which has been confounded with the second stage of that disease, namely, with red hepatization. Where single pulmonary cells have been found dilated in the midst of an undeveloped lobule, the absence of softening, and of the peculiar, congested, humid character of its texture, offers a wide difference between it and the first stage of pneumonia. A portion of lung retaining its fœtal condition allows a little thin, dark, apparently natural blood to escape upon pressure: in the first degree of pneumonia a tolerable quantity of a turbid, bloody fluid, mingled with fibrin, and with a few minute air-vesicles,—in red hepatization, a tenacious dirty-brown reddish,—in grey hepatization, a large proportion of grayish-yellow purulent fluid may be expressed. Atelectasis usually affects both lungs,—pneumonia is, for the most part, confined to one. Finally, the secondary phenomena attendant upon pneumonia, as inflammation of the pleura and of the bronchial mucous membrane, softening of the bronchial glands, fibrinous concrements within the heart's cavities, &c., are wanting in atelectasis. But the peculiar characters of this fœtal condition of the lung are only thus marked during the first few weeks after birth; subsequently, when, as already stated, ulterior changes take place, it becomes extremely difficult to form an exact diagnosis from mere cadaveric inspection.

"Jorg observed atelectasis in children, in whom the first act of breathing had been imperfectly accomplished, either because they were puny and feeble, or because they had been hurried into the world before placental respiration had been altogether suspended, and the necessity for pulmonary respiration become sufficiently potent to stimulate all the muscles of respiration. He therefore concluded, that it was due to the inhaled air not sufficing to effect complete expansion of the lungs; a view corroborated both by the symptoms and course of the affection. This partial introduction of air might be deemed at variance with the physical laws of respiration, inasmuch as the atmospheric pressure must necessarily distend the entire lung equably, not to the exclusion of a lobe, and, still less, to that of a lobule. The objection, however, falls to the ground, when it is considered that the operation of these laws is the result of previous muscular action. Moreover, there is a great analogy between the pathological relations of primal respiration and those of many other affections,—pleurisy, for instance, (see that article,) where the one half of the chest—and especially in partial pleurisy, where certain portions—do not at all share in the movements of the remainder, —and where, again, after the absorption of circumscribed empyema, those very portions collapse and become totally inert, a partial deformity of the chest being the well-known result. We need, therefore, be at no loss to understand how defective breathing may originate in a merely partial activity of the intercostal or other repiratory muscles. It ought to be added, that such portions of the lungs as commonly require several forcible inspirations for their due expansion, are especially prone to remain in the fœtal condition. To conclude it can hardly appear singular that atelectasis should be generally more extensive in the right lung, when we call to mind how the capacity of that half of the thorax is diminished by the great size of the liver in the fœtal state.

"Atelectasis has been confounded with pneumonia by most writers on the diseases of children, as their own publications amply prove; for we find specified, under 'infantile pneumonia,' so much that is peculiar and enigmatical,—so many deviations from the same disease in the adult, as to render it obvious that two maladies of quite a dissimilar nature are brought under one rubric. Even the account they give of the appearances after death, are in many instances conclusive as to the justness of the above assertion; and that without reckoning the previous symptomatic relations,—namely, the frequent absence of true inflammatory phenomena, the want, or irregular accession of fever, and the very slender information derived from percussion and auscultation. The existing difficulties have induced several writers to assume a double form of infantile pneumonia. Rilliet and Barthez distinguish an acute and chronic type: the first occurs in children, somewhat advanced, commencing with the symptoms of catarrh, and resembling in general the pneumonia of adults; the chronic form, on the other hand, assails for the most part new-born infants, and agrees, in symptomatic and anatomical characters, very closely with atelectasis. Thus Billard affirms congestion to be more common in infancy,—true hepatization at a more advanced period; and Heyfelder (l. c. p. 140) confirms the remark made at the hospital for sick children at Paris, namely, that in children beyond the sixth year, pneumonia bears a close affinity to that in the adult. But this division of cases does not remove the difficulty; because, as Gerhard has shown, atelectasis chiefly concerns infants not above twelve months old. Cruse, who very properly rejects the idea of atelectasis being a pneumonia or bronchio-pneumonia, met with genuine inflammation of the lungs in young infants very rarely, and found the after-death appearances to bear a resemblance to those of the disease in adults. His assertion, however, that in quite young infants this inflammation assumes only exceptionally, if ever, the same form as in adults, is in contradiction with experience,—so far at least as the anatomical characters are concerned. Again, the views promulgated with reference to the organic changes affecting the pulmonary texture, further evidence the obscurity and confusion that prevail. Valleix in his otherwise valuable work (p. 195), on the one hand, declares the pneumonia of children (though confounding it with atelectasis) to be identical with that of adults; whilst on the other he is perplexed by certain incongruities, really belonging to atelectasis. His greatest difficul.y is, to account for the '*hepatized*' patches being always hardened, instead of softened, and for their cut surface being smooth, instead of granular. In the new-born child he never met with those secondary results of pneumonia, so constant in adults, and whatever has been encountered of that nature by others, he is forced to ascribe to some other morbific source. He also pointedly adverts to his own experience, as well as to that of others, touching the rarity of pleuritic or bronchitic complication. Billard (l. c. p. 534) expressly seeks to show that this disease essentially differs from the pneumonia of adults. He says: 'the pneumonia of the new-born obviously arises from stasis of the blood in the lungs, the stagnant blood operating as a foreign body,' &c.; and again, 'The cause of the inflammation is purely mechanical, and it is not to be wondered at that the pneumonia is very circumscribed, and indeed limited to the patches originally affected.' Seifert (Die Bronchial-pneumonie der Neugeborenen, &c, 1837,) in a like manner compares the state of the lungs in question with hypostasis, (where, however, softening takes place, and not hardening,) the inflammation being rather of a congestive, venous character. He assumes four grades of bronchio-pneumonia (p. 94); these do not, however, represent the same inflammatory disease in different degrees of advancement, but different forms of one and the same condition, the description of which cannot fail to recall atelectasis to mind. Cruse (l. c. 113) endeavors to convince him that neither hypostasis nor inflammation is present, and hints at the possibility of atelectasis; but, without pursuing the subject any further, refers the pathological changes to bronchitis. Rilliet and Barthez come nearest to the truth, in describing under pneumonia of the new-born a peculiar alteration, namely, carnification. Here the affected portion is commonly situate at the base of the lung; it presents a smooth, compact cut surface, which, on pressure, emits a thin sanguineous fluid; it is sometimes associated with hepatization, and then the lung resembles that of a fœtus that has not yet breathed. The manner in which the disease spreads within the lungs is opposed to the course of pneumonia, but consistent with that of *atelectasis*. Seifert generally found both lungs similarly affected, and Valliex mentions, from researches made by Vernois at the foundling hospital of Paris, that, out 113 cases, there were 100 in which both luugs were simultaneously diseased.

"Unequivocal cases of infantile pneumonia, whether lobar or lobular, such as I have myself examined, and as Kiwisch has published (Oesterreich. Medic. Jahrb. N. F. vol. xxi., Stück 4, p. 534,) afford, on the other hand, the stongest negative grounds for establishing atelectasis as a distinct form. In the great majority of cases, Kiwisch found but one lung affected, the hepatized patches being invariably softened, and rendered cognizable from without, by a dull grayish coloring; conjointly with hepatization the other stages of pneumonia were present, including that of abscess; in every case there was pleuritic effusion more or less copious; bronchitis was seldom noticed,—endocarditis in one case; finally, percussion and auscultation always afforded the ordinary results, whilst the asphyctic and cyanotic phenomena were comparatively trivial.

"In concluding these critical remarks, I consider myself entitled to draw the following inferences: New-born infants are prone to an organic affection of the lungs, altogether distinct from pneumonia, and dependent upon imperfect inspiration after birth, by many pathologists confounded with pneumonia, and by Rilliet and Barthez designated as carnification. The greater number of cases of pulmonary disease occurring at the earliest period of infantile life, and set down as pneumonia, may be looked upon as cases of atelectasis.

This last assertion is, however, to be taken with some reserve,—inasmuch as, in vast laying-in or foundling hospitals (Kiwisch), pneumonia is apt to become epidemic with newborn infants, and, under these circumstances, to attain a numerical preponderance over *atelectasis*."

